# Baohe pill decoction for diarrhea induced by high-fat and high-protein diet is associated with the structure of lactase-producing bacterial community

**DOI:** 10.3389/fcimb.2022.1004845

**Published:** 2022-08-26

**Authors:** Kang Zhou, Na Deng, Xin Yi, Ying Cai, Maijiao Peng, Nenqun Xiao

**Affiliations:** ^1^ College of Pharmacy, Hunan University of Chinese Medicine, Changsha, China; ^2^ College of Chinese Medicine, Hunan University of Chinese Medicine, Changsha, China

**Keywords:** Baohe pill decoction, lactase-producing bacteria, high-fat and high-protein diet, diarrhea, intestinal content

## Abstract

**Background:**

This study investigated the effects of Baohe pill decoction on the diversity and community composition of lactase-producing bacteria in the intestinal contents of mice with diarrhea induced by high-fat and high-protein diet, which provided an experimental basis for the study on the therapeutic mechanism of Baohe pill decoction.

**Materials and methods:**

The Traditional Chinese Medicine Systems Pharmacology (TCMSP), DisGeNET, UniProt, National Center for Biotechnology Information (NCBI), and GeneCards databases were used to collect the potential targets with active ingredients of Baohe pill decoction, diarrhea, and lactase, and then construct correlation networks. Fifteen Kunming mice were randomly divided into the control group (CN), natural recovery group (NR), and Baohe pill decoction treatment group (BHP), with five mice in each group. After constructing a mouse diarrhea model by HFHPD induction, BHP was gavaged with Baohe pill decoction, and the other groups were gavaged with distilled water of equal. The intestinal contents were collected from ileal to jejunal and analyzed using metagenomic sequencing to characterize the intestinal content of lactase-producing bacteria in mice.

**Results:**

The core active ingredients related to diarrhea in Baohe pill decoction were quercetin, luteolin, kaempferol, forsythin, and wogonin. And there was no intersection between the potential targets with the active ingredient of Baohe pill, lactase, and diarrhea. After the intervention of Baohe pill decoction, the Observed species, Chao1 index, and Operational Taxonomic Units (OTU) number increased in BHP (*P* > 0.05), while the Pielous evenness and Shannon index decreased (*P* > 0.05). In Beta diversity, the community structure of the NR was significantly different from CN and BHP (*P* < 0.05), and the community structure of the CN was not significant difference from BHP (*P* > 0.05). Compared to NR, the relative abundance of *Bifidobacterium* and *Amycolatopsis* increased, while the relative abundance of *Lachnoclostridium*, *Sinorhizobium*, *Cedecea*, and *Escherichia* decreased in BHP, but none of the significant differences (*P* > 0.05).

**Conclusion:**

The therapeutic effect of Baohe pill decoction on diarrhea induced by HFHPD does not appear to involve the body’s lactase gene targets directly, but is associated with the change of the construction of lactase-producing bacterial communities.

## 1 Introduction

With the development of animal husbandry and processing technology, the intake of high-fat, high-protein foods has been increasing yearly (Barber et al., 2020). Nevertheless, excessive fat and protein intake are not very beneficial to the human body. A high-fat diet increases the risk of diabetes, hyperlipidemia, and obesity (Stefan, 2020), and a high-protein diet is significantly associated with decrease renal function in patients with myocardial infarction (Esmeijer et al., 2020). Also, too much mono-diet can likewise affect intestinal flora and burden human health.

The intestinal flora is the most complex microbial system in the human body, which plays a vital role in host food digestion, metabolic cycle, and other physiological reactions. Studies have shown that the human body only encodes 17 digestion-related enzyme genes, while the rich enzyme-encoding genes in the intestinal flora complement the host’s ability to digest metabolism (Cantarel et al., 2012). The absence of some carbohydrate-producing active enzyme (CAZyme) strains (e.g. *Prevotella*) can also profoundly alter the absorption of different carbohydrates by the host, thus affecting the physiological or pathological state of the host (Aakko et al., 2020). Notably, other non-carbohydrates can also interfere with the activity of intestinal CAZyme by altering the intestinal flora, such as fat and protein (Ye et al., 2019). Under-digested fats and proteins can affect intestinal health through various pathways, including promoting the expression of pro-inflammatory factors, increasing intestinal mucosal permeability and bacterial translocation, and fermenting to produce toxic metabolites (H_2_S, indole, and ammonia) (Luo et al., 2019; Li et al., 2021; Cai et al., 2022). This further affects intestinal osmolality, causing an imbalance in intestinal homeostasis, decreasing intestinal immunity, and ultimately triggering or exacerbating diarrhea (Sakkas et al., 2020; Zhu et al., 2022). Animal experiments have demonstrated that HFHPD increased the abundance of conditionally pathogenic bacteria *Helicobacter*, *Afipia*, *Clostridium*, and *Phocaeicola*, while decreased the abundance of beneficial bacteria *Lactobacillus* in the intestinal mucosa (Zhu et al., 2022). Remarkably, a variety of intestinal enzyme activities were also significantly decreased after HFHPD intervention, including lactase, which is associated with diarrhea (Shao et al., 2022).

Lactase is an important CAZyme distributed in the intestinal contents and mucosa. If lactase activity is reduced or inhibited, the lactose ingested by the host cannot be metabolized efficiently, predisposing it to osmotic diarrhea. Also, excess lactose will be broken down and converted by intestinal bacteria, producing large amounts of gas and short-chain fatty acids, which can also lead to diarrhea, abdominal pain, and flatulence, clinically known as lactose intolerance (Facioni et al., 2020). Usually, lactase can be obtained by secretion from intestinal epithelial cells and lactase-producing microbes (e.g. Bifidobacterium, *Lactobacillus*, and *Escherichia*) or by exogenous lactase supplementation (He et al., 2017) Lactase-producing bacteria, characterized by fast reproduction and high enzyme production activity, are closely associated with lactase activity and diarrhea. A remarkable example is that adding *Bifidobacterium* and *Lactobacillus* to food alleviates lactose intolerant diarrhea (Oak and Jha, 2019; Popović et al., 2020). Additionally, in the previous studies, we found that antibiotic-induced diarrhea (AAD) not only reduced lactase activity, but also disrupted the community structure of lactase-producing bacteria (Long et al., 2017; Long et al., 2018). While Qiweibaizhu powder, the traditional Chinese medicine (TCM) compound, can treat AAD by regulating the abundance and community structure of lactase-producing bacteria (He et al., 2018; Long et al., 2018). Not only that, the alleviating effect of the probiotic *Debaryomyces hansenii* on AAD was also associated with the promotion of the growth of vital lactase-producing bacteria (He et al., 2019; Wu et al., 2020).

Baohe pill decoction is from the Danxi Xinfa written by Zhu Zhenheng, and is a classic TCM compound for diarrhea caused by improper diet and overeating. It is a combination of seven herbs, namely hawthorn (*Crataegus pinnatifida* Bge), pinellia (*Pinellia ternata* (Thunb.) Breit.), medicated leaven, poria cocos (*Poria cocos* (Schw.)Wolf), tangerine peel (*Citrus reticulata* Blanco), radish seeds (*Raphanus sativus* L.), and forsythia (*Forsythia suspensa* (Thunb.) Vahl), which pharmacologically have the effects of promoting gastric emptying, increasing gastric acid secretion, and anti-ulcer (He et al., 2020). Animal studies reported that Baohe pills could increase the number of Bifidobacteriales, Clostridiales, and Desulfovibrionales, while decrease the number of Bacteroidales, Bacillales, Lactobacillales, Aeromonadales, and Verrucomicrobiales, which in turn reduced the serum total cholesterol, low-density lipoprotein cholesterol, triglyceride levels in rats on high-fat diet (Li et al., 2018). In our previous study, we found that Baohe pill decoction could treat diarrhea induced by HFHPD by promoting the growth of probiotic bacteria *Lactobacillus* and inhibiting the growth of conditionally pathogenic bacteria *Ralstonia* in the intestinal mucosa of mice (Huang et al., 2022). Moreover, Baohe pill decoction can also normalize the activities of lactase rebounded after HFHPD stopped (Shao et al., 2022). The above example indicates that the Baohe pill decoction may regulate lactase activity by altering lactase-producing bacteria, which promotes the improvement of diarrhea symptoms. However, the current Baohe pill decoction and lactase study only focuses on the enzyme activity level.

Based on the microbial functional enzyme gene perspective, we investigated the effect of Baohe pill decoction on lactase-producing bacteria from intestinal contents in diarrhea induced by HFHPD mice. We also explore the relationship between the potential targets with active ingredients of Baohe pill, diarrhea, and lactase with the aid of the network pharmacology, to deeply analyze the mechanism of the efficacy of Baohe pill decoction and promote the clinical application of Baohe pill decoction.

## 2 Materials

### 2.1 Animals

Fifteen SPF-grade Kunming male mice, 18-22 g, were purchased from Hunan Slaccas Jingda Laboratory Animal Co., Ltd (License number: SCXK (Xiang) 2019-0004). Bred in the Animal Experiment Center of Hunan University of Chinese Medicine (relative humidity: 50%~70%, temperature: 23 °C~25 °C). All experiments and procedures involving animals were performed according to the protocols approved by the Institutional Animal Care and Use Committee of the Hunan University of Chinese Medicine.

### 2.2 Medicine

Baohe pill decoction consists of 18 g of *Crataegus pinnatifida* Bge. (Hebei), 6 g of medicated leaven (Sichuan), 9 g of *Poria cocos* (Schw.) Wolf (Hunan), 3 g of *Citrus reticulata* Blanco (Zhejiang), 9 g of *Pineilia ternata* (Thunb.) Breit. (Sichuan), 3 g of *Raphanus sativus* L. (Anhui), and 3 g of *Forsythia suspensa* (Thunb.) Vahl (Shanxi). The above herbs were purchased from the First Affiliated Hospital of Hunan University of Chinese Medicine. Put the above materials into a porcelain jar, add 300 mL of water, boil for 30 min, filter, collect the first filtrate, add 200 mL of water and continue to boil for 30 min, filter, combine the two filtrates, concentrate into a raw drug concentration of 0.28 g/mL of Baohe pill decoction, store at -4 °C for backup (Guo et al., 2022).

### 2.3 Diets

General feed (protein: 20%, fat: 4%) was provided by the Animal Experiment Center of Hunan University of Chinese Medicine. The high-fat and high-protein feeds were (Nestle, protein: 30%, lactose: 0%, fat: 20%), soybean flour (Huiyi, protein: 33%, fat:18%), flour (Huiyi, protein: 13%, fat: 2%), and meatloaf (AnhuiLizheng, 30% protein, 25% fat) were mixed in the ratio of 1:2:2:1. Vegetable oil (Golden Dragonfish). All the above ingredients are lactose free.

### 2.4 Reagents

Proteinase K, TE buffer, lysozyme, Tris-saturated phenol: chloroform: isoamyl alcohol (25:24:1), and acetone were purchased from Beijing Dingguo Biotechnology Co. 0.1 mol/L PBS buffer, 10% SDS, 5 mol/L NaCl, CTAB/NaCl, chloroform: isoamyl alcohol (24:1), 3 mol/L sodium acetate, and 70% anhydrous ethanol, etc. were configured by the laboratory.

## 3 Methods

### 3.1 Network pharmacology analysis of Baohe pill decoction-diarrhea-lactase

#### 3.1.1 Collection of active ingredients and potential targets of Baohe pill decoction

We collected the active ingredients contained in pinellia, medicated leaven, poria cocos, tangerine peel, radish seeds, and forsythia in the TCMSP (http://tcmspw.com/index.php). The TCMSP does not contain entries for the common parts (pulp) of hawthorn and medicated leaven, but there are entries related to hawthorn leaves. According to the literature, the active ingredients of hawthorn leaves are similar to pulp and are often used as a substitute (Lin et al., 2020). Therefore, in this study, hawthorn leaves in the TCMSP were used as the collection target of hawthorn active ingredients, and medicated leaven active ingredients were collected according to the literature reports (Cao et al., 2017; Zhang et al., 2019; Liu et al., 2021). The obtained active ingredients were screened using the drug screening tool provided by TCMSP under the screening conditions of oral bioavailability (OB) ≥ 30% and drug-likeness (DL) ≥ 0.18, and the target proteins of the active ingredients were retrieved by TCMSP. The target proteins were then entered into the UniProt (https://www.uniprot.org/) database, and its protein-acting gene names were calibrated, with the population limited to “Homo sapiens”.

#### 3.1.2 Collection of potential targets for diarrhea

We searched the DisGeNET database (https://www.disgenet.org/) and GeneCards database (https://www.genecards.org/) for relevant targets using the keyword “diarrhea”. After removing targets with a systematic score < 0.01 from the DisGeNET database and targets with a systematic score < 35 from the GeneCards database, the remaining targets were combined, de-duplicated, and unified in format to obtain potential targets for diarrhea.

#### 3.1.3 Collection of potential targets for lactase

We searched the NCBI (https://www.ncbi.nlm.nih.gov/) database for relevant targets using the keyword “lactase”. The results were further screened under the “Homo sapiens” condition to obtain potential lactase targets.

#### 3.1.4 Construction of the Baohe pill decoction-active ingredient-lactase-diarrhea correlation network

We imported the collected relevant targets with active ingredients of Baohe pill decoction, diarrhea, and lactase into R language (v4.1.1, https://www.r-project.org/) and Cytoscape (v3.9.1, https://cytoscape.org/). The Venn diagram was constructed using the Venn package in R, and the Baohe pill potential active ingredient-lactase-diarrhea network was constructed using Cytoscape.

### 3.2 Animal experimental process

**Figure 1:**
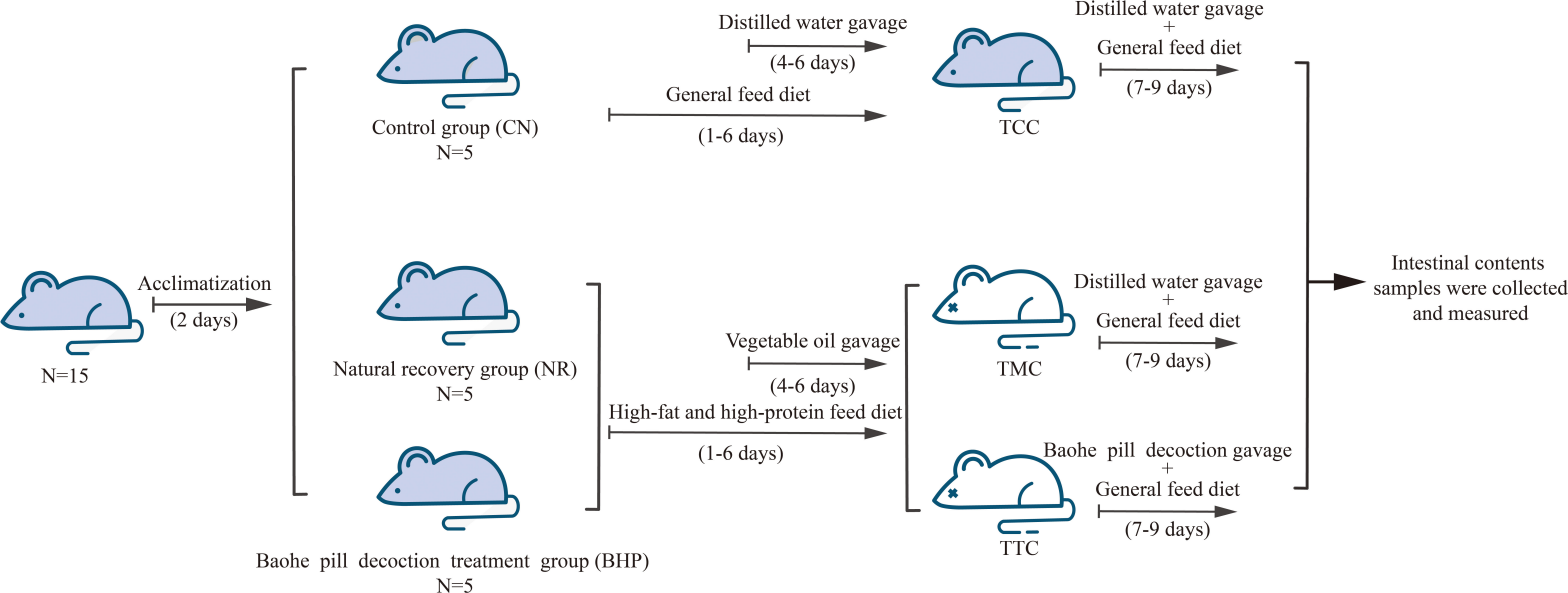
Animal experiment flow.

15 mice were randomly divided into the control group (CN), natural recovery group (NR), and Baohe pill decoction treatment group (BHP) after 2 d of adaptive feeding. 5 mice in each group, 5 in 1 cage. The modeling method refers to literature (Shao et al., 2022), and the study flow is shown in **Figure 1**. NR and BHP mice were given HFHPD, and gavage with vegetable oil (0.4 mL/time, 2 times/d for 3 d) starting from the 4th day. During this period, CN were given a general diet and gavaged with distilled water instead of vegetable oil on d 4. After 6 d of HFHPD intervention, the modeling factor was stopped, BHW was gavage with Baohe pill decoction at a 6.63 g/(kg·d) dose twice a day for 3 d, and CN and NR were given gavage distilled water of equal amount.

### 3.3 Extraction of Intestinal content

All mice were sacrificed by the cervical dislocation method, and then the intestine was dissected under aseptic conditions. The contents of the jejunum to ileum segment were taken with sterile forceps, placed in centrifuge tubes, labeled and weighed, and stored at -80 ℃ for backup (Shao et al., 2020).

### 3.4 DNA extraction

After sample pretreatment, total bacterial DNA was obtained by acetone washing, lysozyme wall breaking, proteinase K denaturation, SDS lysis, CTAB treatment, and phenol/chloroform extraction, with detailed steps according to the reference (Hui et al., 2020).

### 3.5 PCR Amplification

Amplification using our previously reported lactase primers (Long et al., 2017), i.e., upstream primer: 5′-TRRGCAACGAATACGGSTG-3′ and downstream primer: 5′-ACCATGAARTTSGTGGTSARCGG-3′. PCR amplification system was as follows: 0.25 μL of Q5 high-fidelity DNA polymerase, 5 μL of 5 × Reaction Buffer, 5 μL of 5 × High GC Buffer, 0.5 μL of dNTP (10 mM), 1 μL of template DNA, 1 μL of upstream primer (10 uM), 1 μL of downstream primer (10 uM), and 11.25 μL of ultrapure water. After the above conditions were configured, the PCR instrument was pre-denatured at 98 °C for 30 s and entered the amplification cycle. Firstly, the template was kept at 98 °C for 15 s to denature fully, then kept at 46 °C for 30 s to anneal, and then kept at 72 °C for 30 s to fully extend the primers to form one cycle. The cycle was repeated 32 times, then extended for 5 min at 72 °C and stored at 4 °C. The sequencing was performed by Shanghai Personalbio Technology Co.

### 3.6 Bioinformatics analysis

#### 3.6.1 OTU division and classification level annotations

The resulting sequences were spliced, de-duplicated, filtered, and divided into OTUs with a 97% similarity threshold using Vsearch (v2.13.4_linux_x86_64) and Cutadapt (v2.3) software. The divided OTU (Blaxter et al., 2005) representative sequences were compared with the NCBI database using blastn on Qiime2 (2019.4, https://qiime2.org/) software, and then The brocc.py script was called to obtain OTU species annotation information.

#### 3.6.2 Alpha diversity analysis

Chao1, Observed species, Shannon, Simpson, Pielous evenness, Goods coverage index and Shannon rarefaction curves were calculated using Qiime2 and visualized with R (Li et al., 2019).

#### 3.6.3 Beta diversity analysis

Beta diversity can represent the differences in the composition of different communities. Principal coordinate analysis (PCoA) and Non-metric multidimensional scaling (NMDS) were performed by Qiime2 to visualize community differences (Ramette, 2007), and the PERMANOVA (Permutational multivariate analysis of variance) test was used to represent the differences between communities, and *P* < 0.05 was considered significant.

### 3.7 Statistical analysis

The data were analyzed using IBM SPSS (v25.0) software, and the experimental results data were expressed as mean ± standard deviation, and one-way ANOVA or Kruskal-Wallis test was used according to whether the data were normally distributed and the variance was consistent. *P* < 0.05 was considered a significant difference.

## 4 Results

### 4.1 Active ingredients and corresponding potential targets of the Baohe pill decoction

After collecting the effective active ingredients of Baohe pill decoction by TCMSP search and literature search methods, 44 active ingredients meeting OB ≥ 30% and DL≥ 0.18 were obtained (**Table 1**). Among them, there were 2 hawthorn, 10 pinellia, 6 poria cocos, 14 forsythia, 4 tangerine peel, 1 radish seed, 1 medicated leaven, 1 total of pinellia and hawthorn, 1 total of pinellia and hawthorn, 1 total of tangerine peel, hawthorn and radish seed, 1 total of forsythia and medicated leaven, 2 total of forsythia, medicated leaven and hawthorn. The corresponding potential targets of each active ingredient were obtained in TCMSP, and a total of 273 potential target genes were obtained after comparison and de-duplication by the Uniprot database.

**Table 1:** Baohe pill decoction active ingredient.

	Mol ID	Molecule Name	OB (%)	DL (%)
pin+for1	MOL000358	beta-sitosterol	36.91	0.75
pin+haw1	MOL000449	Stigmasterol	43.83	0.76
pinellia1	MOL001755	24-Ethylcholest-4-en-3-one	36.08	0.76
pinellia2	MOL000519	coniferin	31.11	0.32
pinellia3	MOL002670	Cavidine	35.64	0.81
pinellia4	MOL002714	baicalein	33.52	0.21
pinellia5	MOL002776	Baicalin	40.12	0.75
pinellia6	MOL003578	Cycloartenol	38.69	0.78
pinellia7	MOL005030	gondoic acid	30.70	0.20
pinellia8	MOL006936	10,13-eicosadienoic	39.99	0.20
pinellia9	MOL006957	(3S,6S)-3-(benzyl)-6-(4-hydroxybenzyl)piperazine-2,5-quinone	46.89	0.27
pinellia10	MOL006967	beta-D-Ribofuranoside, xanthine-9	46.72	0.21
radish seed1	MOL010672	icosa-8,11,14-trienoic acid methyl ester	44.81	0.23
rad+tan+haw1	MOL000359	sitosterol	36.91	0.75
tangerine peel1	MOL005100	5,7-dihydroxy-2-(3-hydroxy-4-methoxyphenyl)chroman-4-one	47.74	0.27
tangerine peel2	MOL004328	naringenin	59.29	0.21
tangerine peel3	MOL005828	nobiletin	61.67	0.52
tangerine peel4	MOL005815	Citromitin	86.90	0.51
poria cocos1	MOL000282	ergosta-7,22E-dien-3beta-ol	43.51	0.72
poria cocos2	MOL000296	hederagenin	36.91	0.75
poria cocos3	MOL000279	Cerevisterol	37.96	0.77
poria cocos4	MOL000275	trametenolic acid	38.71	0.80
poria cocos5	MOL000273	(2R)-2-[(3S,5R,10S,13R,14R,16R,17R)-3,16-dihydroxy-4,4,10,13,14-pentamethyl-2,3,5,6,12,15,16,17-octahydro-1H-cyclopenta[a]phenanthren-17-yl]-6-methylhept-5-enoic acid	30.93	0.18
poria cocos6	MOL000283	Ergosterol peroxide	40.36	0.81
forsythia1	MOL000173	wogonin	30.68	0.23
forsythia2	MOL000522	arctiin	34.45	0.84
for+med1	MOL000006	luteolin	36.16	0.25
forsythia3	MOL003305	Phillyrin	36.40	0.86
for+med+haw1	MOL000422	kaempferol	41.88	0.24
forsythia4	MOL003347	hyperforin	44.03	0.61
for+med+haw2	MOL000098	quercetin	46.43	0.28
forsythia5	MOL003290	(3R,4R)-3,4-bis[(3,4-dimethoxyphenyl)methyl]oxolan-2-one	52.30	0.48
forsythia6	MOL003295	(+)-pinoresinol monomethyl ether	53.08	0.57
forsythia7	MOL003308	(+)-pinoresinol monomethyl ether-4-D-beta-glucoside_qt	61.20	0.57
forsythia8	MOL000211	Mairin	55.38	0.78
forsythia9	MOL003283	(2R,3R,4S)-4-(4-hydroxy-3-methoxy-phenyl)-7-methoxy-2,3-dimethylol-tetralin-6-ol	66.51	0.39
forsythia10	MOL000791	bicuculline	69.67	0.88
forsythia11	MOL003322	FORSYTHINOL	81.25	0.57
forsythia12	MOL003370	Onjixanthone I	79.16	0.30
forsythia13	MOL003306	ACon1_001697	85.17	0.57
forsythia14	MOL003330	(-)-Phillygenin	95.04	0.57
medicated leaven 1	MOL007424	Artemisinin	49.88	0.31
hawthorn1	MOL000354	isorhamnetin	49.60	0.31
hawthorn2	MOL000073	ent-Epicatechin	48.96	0.24

### 4.2 Diarrhea and lactase potential targets

The diarrhea targets obtained in the DisGeNET and GeneCards databases were 632 and 5721, respectively, and a total of 3399 potential targets were obtained after the screening, merging, and de-duplication. The number of lactase-potential targets obtained in the NCBI database was 1651, and 12 lactase targets were obtained after screening.

### 4.3 Baohe pill decoction-active ingredient-lactase-diarrhea network analysis

**Figure 2:**
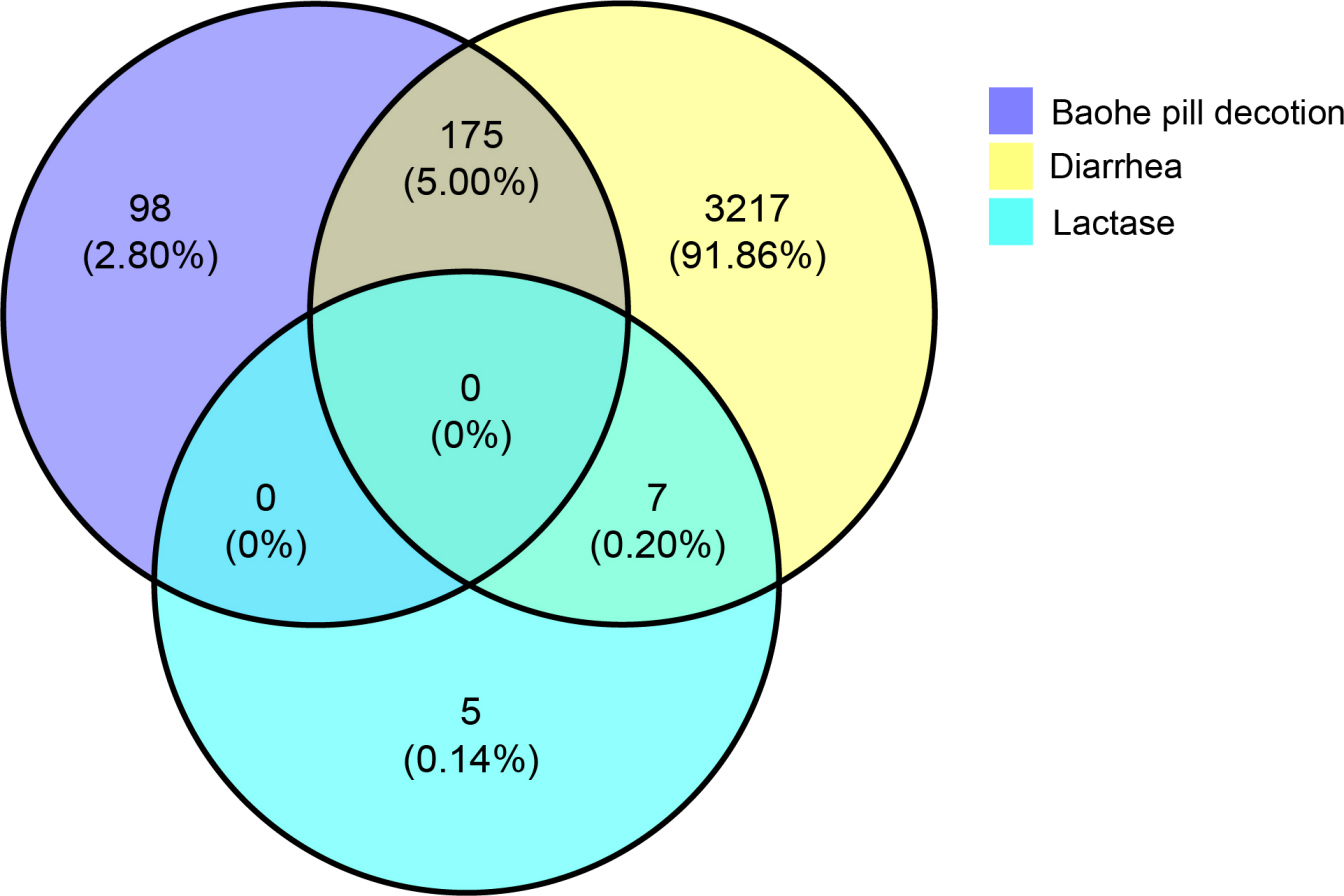
Venn diagram of the active ingredient of Baohe pill decoction -lactase-diarrhea. Each circle represents a group, the overlapping area between the circles refers to the common targets between the groups, and the number of each block indicates the number of targets contained in the area.

**Figure 3:**
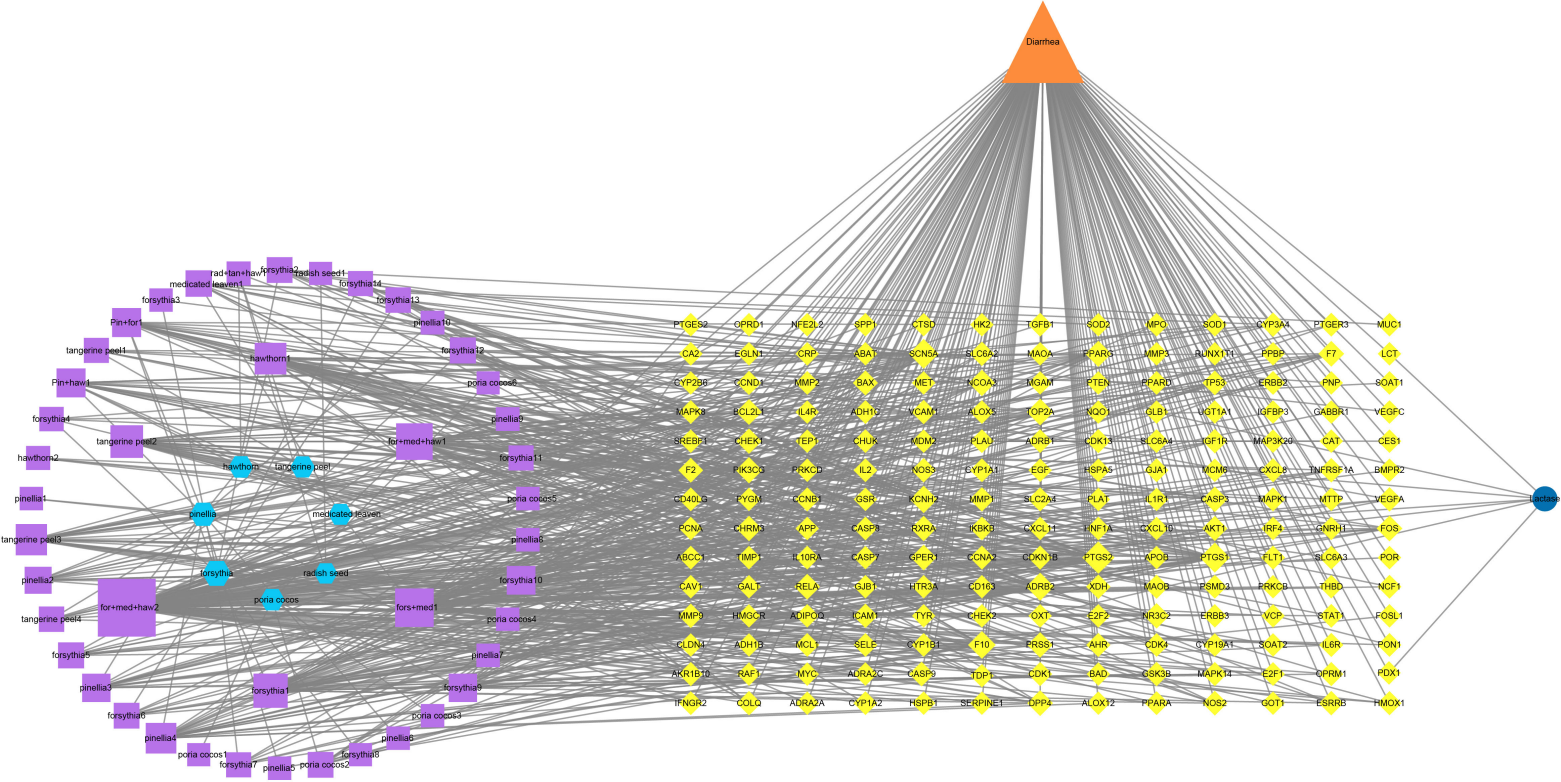
Baohe pill-active ingredient-lactase-diarrhea correlation network. The light blue hexagon is the 7 herbs composed of Baohe pill, the purple square is the active ingredient, the yellow diamond is the potential target, the orange triangle is diarrhea, and the dark blue circle is lactase. The connection line between the nodes represents the corresponding relationship between the two.

As seen in **Figure 2**, the intersection targets of Baohe pill decoction and diarrhea are 175, the intersection targets of diarrhea and lactase are 7, and the intersection targets of the three are 0. From the perspective of the total number of human lactase targets, this means that there is a relatively close correlation between lactase and diarrhea, but there is no direct interaction between the lactase targets and the active ingredient of Baohe pill decoction. The active ingredient of the Baohe pill decoction, lactase and diarrhea intersecting target, and Baohe pill decoction and diarrhea intersecting target were input into Cytoscape to obtain the Baohe pill decoction-active ingredient-lactase-diarrhea correlation network (**Figure 3**). There are 235 nodes and 774 edges in the network. The degree value of each node in the network was obtained by the network analysis tool in Cytoscape. The higher the degree value indicates that the more points the ingredient is connected to, the higher the possibility that the ingredient is a core ingredient. The five highest degree values among the active ingredients of the Baohe pill were quercetin (degree: 107, forsythia, medicated leaven, and hawthorn), luteolin (degree: 48, forsythia and medicated leaven), kaempferol (degree: 42, forsythia, medicated leaven, and hawthorn), phillyrin (degree: 42, forsythia, medicated leaven, and hawthorn), wogonin (degree: 35, forsythia), involving a total of 124 diarrhea targets. From the target point of view, the five targets with the highest degree values were PTGS2 (degree: 29), PTGS1 (degree: 22), SCN5A (degree: 21), F10 (degree: 15), and ADRB2 (degree: 16). It indicates that quercetin, luteolin, kaempferol, phillyrin, and wogonin are the most likely core ingredients of Baohe pill decoction for diarrhea.

### 4.4 General features

After 6 d of HFHPD intervention, CN mice had smooth and flat fur, good mental status, and brown wheat-shaped stools. NR and BHP mice had loose fur, reduced activity, soft texture of stool, easily deformed and stuck to the forceps when picked up with forceps, and half of the stool was in the form of a thick paste and stuck to the tail and perianal area. After 3 d gavage of Baohe pill decoction, the fur and mental condition of BHP mice gradually returned to normal, the texture of stool changed from thick paste or soft rotten to normal wheat grain shape, and the perianal area and tail were clean and free of foreign matter. NR also approached the condition of CN, but individual mice were still mentally lethargic and inactive.

### 4.5 Effect of Baohe pill decoction on the number of OTUs of lactase-producing bacteria in the intestinal contents of HFHPD mice

**Figure 4:**
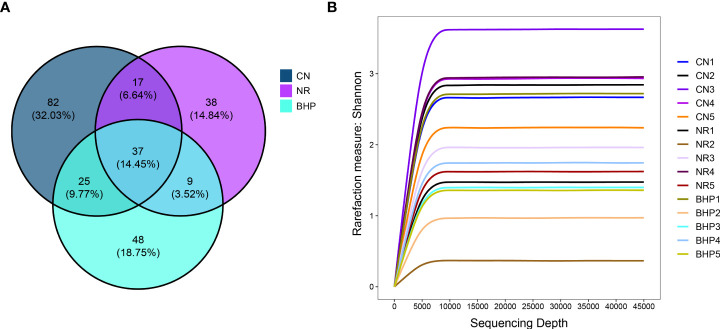
**(A)** Venn diagram of the number of OTUs distribution of lactase-producing bacteria from the intestinal contents in each group. Each circle represents a group, the overlapping area between the circles refers to the common OTUs between the groups, and the number of each area indicates the number of OTUs contained in the area. **(B)** Rarefaction curve of the lactase-producing bacteria from the intestinal contents in each group. The smoothness of the rarefaction curve reflected the effect of sequencing depth on the diversity of observed samples. The flatter the curve, the more indicating that the sequencing results were sufficient to reflect the diversity contained in the current sample. CN, control group; NR, natural recovery group; BHP, Baohe pill decoction treatment group.

The number of totals and unique OTUs among different groups can be visualized by the Venn diagram. As shown in **Figure 4**, the number of OTUs unique to the CN, NR, and BHP groups was 82, 38, and 48, respectively, and the total OTUs was 37. The number of OTUs in NR was lower than that in CN, while compared with NR, the number of OTUs in BHP and the number of OTUs shared with CN were increased. The results indicated that HFHPD decreased the taxonomic units number of lactase-producing bacteria in the intestinal contents of mice, and the number rose after treatment with Baohe pill decoction.

### 4.6 Effect of Baohe pill decoction on Alpha diversity of lactase-producing bacteria in the intestinal contents of HFHPD mice

Alpha diversity in microbial communities can be measured by indices such as Shannon, Chao1, and Simpson. The larger Chao1 and Observed specie indices, the greater the number of species and the higher the richness of the community. Simpson index size reflects the community’s diversity, and the larger the value, the higher the community’s diversity. Pielou eveness index focuses on the evenness of the community, and the higher the value, the more even the community. The Shannon index combines the richness and evenness of the community. The Goods coverage index is related to the species coverage of the detection results, and the higher the value, the higher the proportion of detected species in the total species of the sample.

**Figure 5:**
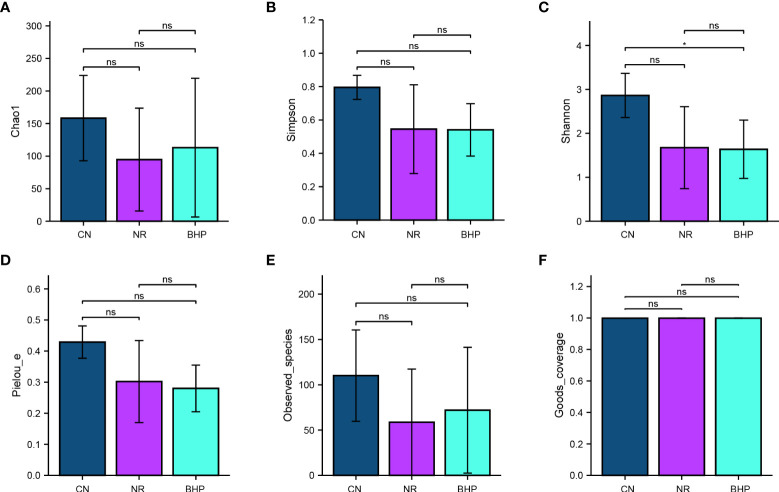
Alpha diversity index of the lactase-producing bacteria from the intestinal contents in each group. **(A)**: Chao1. **(B)**: Simpson. **(C)**: Shannon. **(D)**: Pielous evenness. **(E)**: Observed specie. **(F)**: Goods coverage. Chao1 and Observed species indices represent community richness, Shannon and Simpson indices represent community diversity, Pielou’s evenness index represents community evenness, and Good’s coverage index represents the percentage of detected species in the sample. *: *P* < 0.05. CN, control group; NR, natural recovery group; BHP, Baohe pill decoction treatment group ns: P ≥ 0.05.

The Shannon rarefaction curve (**Figure 4B**) shows that each sample’s species diversity and richness have leveled off with increasing depth, indicating that there is little gain from continuing to increase the sequencing depth. From **Figure 5**, The Goods coverage indexes of CN, NR, and BHP were all higher than 99.9%, indicating that the number of species covered by sequencing was sufficient and the sequencing results were all representative of the actual situation of the microbial communities in the samples. The results of the remaining 5 diversity indices showed that CN had the highest richness and diversity, followed by BHP and NR. Compared with NR, Observed species, Chao1 index increased in BHP, while Pielous evenness and Shannon index decreased. However, there was no significant difference (*P* > 0.05) in all data comparisons except in the Shannon index, where CN and BHP were significantly different (*P* < 0.05). The results showed that HFHPD-induced diarrhea reduced the diversity, richness, and evenness of lactase-producing bacteria, and Baohe pill decoction had a restoring effect on the richness of lactase-producing bacteria, but diversity and evenness did not improve.

### 4.7 Effect of Baohe Pill decoction on Beta diversity of lactase-producing bacteria in the intestinal contents of HFHPD mice

**Figure 6:**
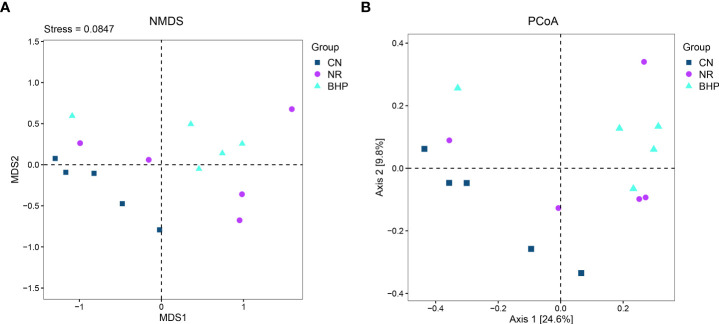
Beta diversity of the lactase-producing bacteria from the intestinal contents in each group. **(A)** NMDS (Based on Jaccard distance). **(B)** PCoA (Based on Jaccard distance). By PERMANOVA test, the community structure of the NR was significantly different from CN and BHP (*P* < 0.05), and the community structure of the CN was not significant difference from BHP *(P* > 0.05). In the graph, each point represents a sample, and samples of the same color are from the same group. The greater the difference between every two points, the greater the difference between the two samples. FCM, control group; FMM, model group; CN, control group; NR, natural recovery group; BHP, Baohe pill decoction treatment group.

Beta diversity analysis could quantify the differences in community structure among different samples. In NMDS analysis, the smaller the Stress value is, the more accurately it reflects the actual distribution of the sample data, and it is generally considered that the NMDS analysis results are more reliable when it is less than 0.2. In the NMDS (**Figure 6A**) analysis, the Stress value was 0.08, which indicates that the current analysis results are well represented. CN and BHP can be more clustered individually. CN is mainly distributed in the third quadrant, BHP is mainly distributed in the first quadrant, and NR is relatively chaotic. The results of the PCoA analysis (**Figure 6B**) were similar to the NMDS analysis. The PERMANOVA test showed the community structure of the NR was significantly different from CN and BHP (*P* < 0.05), and the community structure of the CN was not significant difference from BHP (*P* > 0.05). It indicates that HFHPD significantly changed the composition structure of lactase-producing bacteria in the intestinal contents of mice, and Baohe pill decoction significantly modulated the structure of lactase-producing bacteria in the intestinal contents of HFHPD mice.

### 4.8 Effect of Baohe pill decoction on the taxonomic composition of lactase-producing bacteria in the intestinal contents of HFHPD mice

**Figure 7:**
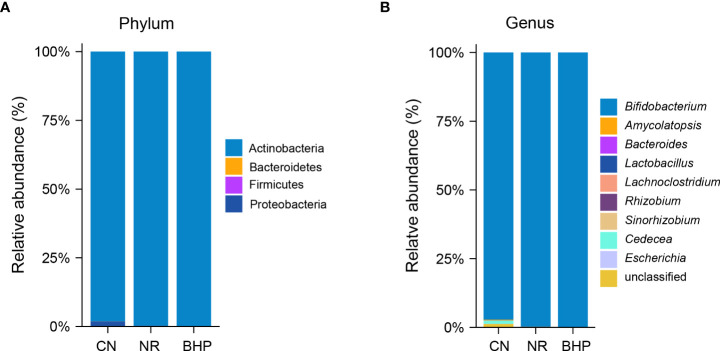
Taxonomic composition of the lactase-producing bacteria from the intestinal contents in each group. **(A)** Phylum level. **(B)** Genus level. CN, control group; NR, natural recovery group; BHP, Baohe pill decoction treatment group.

As seen in **Figure 7A**, Actinobacteria were the dominant phylum of lactase-producing bacteria in mouse intestinal contents, accounting for approximately 98% of lactase-producing bacteria in all groups of mice. In addition, Bacteroidetes were distributed only in CN, and Firmicutes were distributed only in CN and NR. At the genus level (**Figure 7B** and **Table 2**), the lactase-producing groups of CN mouse intestinal contents were mainly composed of *Bifidobacterium* (96.53%), *Cedecea* (1.69%), *Amycolatopsis* (0.34%), *Escherichia* (0.01%), unclassified (1.41%) and other low abundance taxa composition (0.02%). Compared to NR, the relative abundance of *Amycolatopsis* and *Bifidobacterium* increased, while *Lachnoclostridium*, *Sinorhizobium*, *Cedecea*, and *Escherichia* decreased in BHP (*P* > 0.05).

**Table 2:** Relative abundance of lactase-producing bacteria in each group at the genus level.

	CN	NR	BHP
*Bifidobacterium*	0.965365 ± 0.057772	0.996772 ± 0.003149	0.998922 ± 0.000648↑
*Amycolatopsis*	0.003357 ± 0.005014	0	0.000008 ± 0.000015↑
*Bacteroides*	0.000009 ± 0.000018	0	0
*Lactobacillus*	0.000027 ± 0.000054	0	0
*Lachnoclostridium*	0	0.000004 ± 0.000005	0↓
*Rhizobium*	0	0.000280 ± 0.000451	0.000332 ± 0.000404↑
*Sinorhizobium*	0	0.001600 ± 0.003088	0↓
*Cedecea*	0.016979 ± 0.033900	0.000105 ± 0.000157	0.000003 ± 0.000005↓
*Escherichia*	0.000183 ± 0.000367	0.000004 ± 0.000009	0↓
unclassified	0.014080 ± 0.019868	0.001235 ± 0.002161	0.000736 ± 0.000855↓

Compared with NR, ↑ indicates an increase in strain abundance, ↓ indicates a decrease in strain abundance. CN, control group; NR, natural recovery group; BHP, Baohe pill decoction treatment group.

## 5 Discussion

By secreting abundant specific enzymes, intestinal flora can degrade and modify ingested substances and produce new biosignaling molecules, which in turn are involved in the pathological and physiological activities of the body systems (Zhou et al., 2019). For example, the human liver can inactivate the toxic active metabolite SN-38 produced by the anticancer drug irinotecan, converting it to the non-toxic SN-38-G. However, in the colon, microbial β-glucuronidase can reactivate SN-38-G to SN-38, triggering irinotecan dose-limiting diarrhea (Ervin et al., 2019). In TCM, sennosides, as precursor substances for rhubarb to exert diarrheal effects, also need to be transformed into their pharmacologically active form by the action of microbial reductases (Quan and Huan, 2021). Besides, the therapeutic mechanism of some TCM compounds for diarrhea is related to the regulation of lactase activity, but further studies on the relationship between drugs and microbial enzyme genes are relatively rare (Wu and Tan, 2021). Therefore, the analysis of lactase-producing enzyme strains is beneficial as biomarkers for in-depth dissection of disease pathological mechanisms, and is significant for the study of new potential targets for drug therapy.

In the network pharmacology section (**Figure 3**), we screened out five top active ingredients associated with diarrhea (quercetin, luteolin, kaempferol, phillyrin, and wogonin). Among them, quercetin could improve lactase activity and appears to be associated with lactase-producing bacteria. For example, an animal experiment showed that lactase activity of rat spleen increased by 58% after feeding quercetin (Chougala et al., 2012). Another animal experiment showed that quercetin significantly decreased the abundance of *Escherichia coli* in the cecum of broiler chickens, while significantly increased the abundance of *Lactobacillus*, *Bifidobacterium*. Moreover, *in vitro* experiment, quercetin damaged *Escherichia coli* cell wall, but the lactase activity produced by *Escherichia coli* was increased (Wang et al., 2018). Luteolin can modulate lipid metabolism disorders and reduce the elevated *Lactobacillus* and *Prevotella_9* abundance in rats with ulcerative colitis (Li et al., 2021). Kaempferol alleviating effect on experimental colitis is associated with an increased Firmicutes/Bacteroidetes ratio and the abundance of beneficial bacteria *Prevotellaceae* and *Ruminococcaceae* (Qu et al., 2021). The above demonstrated that the main active ingredients of the Baohe pill decoction associated with diarrhea all have good modulating effects on the intestinal flora. More notably, the human lactase targets did not intersect with the active ingredient targets (**Figure 2**). This means that after entering the intestinal tract, the active ingredients of Baohe pill decoction did not directly affect the human body’s lactase gene target. It is more likely to regulate the level of lactase activity by affecting lactase-producing microbe or by conversion through other metabolic pathways. Based on these results, our mouse experiments further validated the effect of Baohe pill decoction on lactase-producing bacteria.

In the experimental results, the diversity and evenness of lactase-producing bacteria decreased, and the abundance increased after the treatment of Baohe pill decoction (**Figure 5**), which led to a greater difference in the abundance proportion among individual lactase-producing bacteria. This was also evidenced by the higher relative abundance of the dominant bacteria after the intervention of Baohe pill decoction. Moreover, the effect of both HFHPD and Baohe pill decoction on the Alpha diversity of lactase-producing bacteria was not significant (*P* > 0.05), which may be related to the gradual natural recovery of intestinal contents bacteria after the cessation of HFHPD. The Beta diversity showed that Baohe pill decoction recovered lactose-producing bacterial structure (*P* < 0.05). Microbial lactase has good functional gene diversity, leading to variability in microbial lactase activity from different sources, and the composition of different lactase-producing bacteria has a greater impact on lactase activity. Simultaneously, in our previous studies on lactase-producing bacteria from intestinal contents, the therapeutic effect of Qiweibaizhu powder is also mainly reflected in the promotion of specific lactase-producing bacteria (e.g. *Acidovorax* sp. KKs102), rather than affecting the diversity of lactase-producing bacteria (He et al., 2018). This means that the modulating effect on the structure of the lactase-producing bacterial community is a critical factor in the therapeutic efficacy of Baohe pill decoction.

In the taxonomic composition (**Table 2**), the relative abundance of *Bifidobacterium* in the three groups was ranked as CN < NR < BHP. *Bifidobacterium* is a recognized probiotic in the intestine with an essential role in regulating immune function, maintaining intestinal homeostasis, and defending against inflammatory diseases. And which is also one of the main sources of microbial lactase (Lim and Shin, 2020). However, Brandao et al. (Brandao Gois et al., 2022) found a positive correlation between *Bifidobacteria* abundance with dairy intake and adverse effects in lactose intolerant patients. We speculate that the harmful fermentation products of high protein and the high fat-induced increase in endotoxin may have acted similarly to the effect of lactose in promoting the growth of *Bifidobacterium*, i.e., stimulating adaptive changes in the intestinal contents flora. Enzymes are proteins encoded by functional genes, and protein activity is closely related to their structure and modification. Lactase gene expression and protein modifications can be altered under different microcosmic environments (Vera et al., 2020). Meanwhile, enzyme activity is affected by pH, and HFHPD can also change intestinal pH (Wang et al., 2021; Shao et al., 2022), resulting in the normal expression of the lactase gene or activation of zymogen may be hindered by the deterioration of the intestinal environment caused by HFHPD. And the further increase in the relative abundance of *Bifidobacterium* by Baohe pill decoction appears to relate to the ameliorative effect on the intestinal environment. For instance, You et al. (2021) intervened the ulcerative colitis mice with Baohe pill decoction. They found that Baohe pill decoction could improve the abundance of immune regulatory flora and repair the damaged intestinal mucosa. *Lactobacillus* is one of the most widely used lactase-producing bacteria, and the decrease in the relative abundance of *Lactobacillus* in NR indicates the harmful effect of HFHPD on intestinal lactase-producing bacteria. Studies have shown that Baohe pill decoction promotes the abundance of Lactobacillus in the intestinal mucosa (Zhu et al., 2022), but Baohe pill decoction does not affect the abundance of Lactobacillus in the intestinal contents. The reason may be related to the differences in intestinal contents and mucosal flora function. For instance, it has been shown that the composition of the intestinal mucosal flora is more sensitive than the intestinal contents in repeated stress-related diarrhea (Zhang et al., 2021).

Finally, there are still some limitations in this study, such as the network pharmacology part is only based on database theory, and the experimental part needs further validation of lactase gene expression.

## 6 Conclusion

In this study, the effects of Baohe Pill decoction on disease-related microbial functional enzyme genes were investigated by network pharmacology and metagenomic sequencing, which will also provide a basis for further study the mechanism of therapeutic Baohe pill decoction at the gene level. The results show that the Baohe pill decoction does not appear to have direct effects on the body’s lactase targets, and its therapeutic effect on diarrhea induced by HFHPD was related to lactase-producing bacteria. Specifically, the Baohe pill decoction changed the community structure of lactase bacteria in intestinal contents and regulated the diversity and relative abundance of lactase bacteria to a certain extent.

## Data availability statement

The datasets presented in this study can be found in online repositories. The names of the repository/repositories and accession number(s) can be found below: https://www.ncbi.nlm.nih.gov/, PRJNA861941

## Ethics statement

The animal study was reviewed and approved by Animal Care and Use Committee of the Hunan University of Chinese Medicine.

## Author contributions

KZ: data analysis and writing the original draft. ND and YC: review and editing. KZ and XY: performing animal experiments. MP and NX: project administration, review, and funding acquisition. All authors contributed to the article and approved the submitted version.

## Funding

This research was financially supported by the Natural Science Foundation of Hunan Province (No. 2020JJ4468) and the Hunan Chinese Medicine First-Class Discipline Project (2018).

## Acknowledgments

We thank the Natural Science Foundation of Hunan Province (No. 2020JJ4468) and the Hunan Chinese Medicine First-Class Discipline Project (2018) for the financial support of this study.

## Conflict of interest

The authors declare that the research was conducted in the absence of any commercial or financial relationships that could be construed as a potential conflict of interest.

## Publisher’s note

All claims expressed in this article are solely those of the authors and do not necessarily represent those of their affiliated organizations, or those of the publisher, the editors and the reviewers. Any product that may be evaluated in this article, or claim that may be made by its manufacturer, is not guaranteed or endorsed by the publisher.
